# Supplementary material for: Association between microbial water quality, sanitation and hygiene practices and childhood diarrhea in Kersa and Omo Nada districts of Jimma Zone, Ethiopia
Source: PLoS One. 2020 Feb 19;15(2):e0229303. doi: 10.1371/journal.pone.0229303 (PMC7029864; doi:10.1371/journal.pone.0229303)
Supplement: S1 File — Questionnaire used for data collection. (PDF) [file pone.0229303.s001.pdf]

English questionnaire

Household Identification Code______________ Name of Kebele______________

Verbal Consent Letter

Dear interviewee, I extend my greeting to you. I / we are here to collect health related data for the purpose of research from Addis Ababa University, Ethiopian Institute of Water Recourses. The aim of this study is to assess the association between microbial water quality, sanitation and hygiene interventions and childhood diarrhea in Kersa and Omo Nadda districts of Jimma Zone, Ethiopia. We are requesting your permission to participate in an interview on issues related to the microbial water quality and effectiveness of sanitation and hygiene practices on childhood diarrhea reduction.

This information will help the policy makers and other responsible bodies as background to improve the health status of the children related to childhood diarrhea.

We assure you that whatever information you provide will only be used for the purpose of this research and will not be made available to anyone outside of the research team.

Your willingness and support to people respond the interview is very much appreciated. We also assure that the interview process will not bring any harm to you and your family. It is also your right to withdraw any time from the process when your feeling is uncomfortable with it.

Please make (X) mark to indicate the respondents' decision regarding participation in the study. The purpose of the study and confidentiality procedures has been explained to me and I on my own consent: a) Agree _____ b) Disagree_____

Name of interviewer------------------------- sign---------------------

Date --Time started: --------------------- Time ended: -------------------

**S. No**	**Questions**	Alternatives			**Skips**	**Mark /code**
	**Assets and other housing characteristics**					
101.	This house is……? (Tick one)	1. Owned 2. Rented<br>3. Other___________				
102.	Does your household have:		Yes	No		
		• Electricity				
		• Radio				
		• Television				
		• Refrigerator				
		• Table				
		• chair				
		• Mobile phone				
		• Bed with cotton/sponge/spring mattress				
		• Any type of cattle	If yes, No ----			
103.	What type of fuel does your household mainly use for cooking?	1. electricity or gas<br>2. Wood<br>3. Others -------------------------------				
104.	Type of lighting	1. electricity or gas				

		2. candle or wood		
**105.**	What is the main material of the floor in your household?	1. concrete or wood<br>2. Earth/sand/dung<br>3. Other ------------------------------		
**106.**	What is the main material of the roof in your household?	1, Corrugated iron/metal<br>2, Thatch/Leaf/Mud<br>3, Others ---------------------------------		
**107.**	Does your household have farming land	1. Yes, ⇒ write in hectare -------<br>2. No		
**108.**	Does any member of this household have a bank or microfinance saving account?	1 Yes 2 No		
**Socio-demographic characteristics**				
109.	How many people live in this household?	------------		
110.	Number of under five in the households?	-------------		
111.	Age of mother/ caregiver of the child (years)	----------------		
112.	Religion of mother/ caregiver of the child	1 Muslim 2 Orthodox 3 Protestants		
113.	Ethnicity of mother/ caregiver of the child	1 Oromo 2 kafa 3 Yem 4 Others		
114.	Marital status	1. Single 2. Married<br>3. Divorced/Separated 4. Widow		
115.	Educational status of the mother/ caregiver of the child	1. no/lack of formal education 2. Primary 3. Secondary<br>4. College /University and above		
116.	Occupation of mother/caregiver of the child	1. House wife 2. Merchant<br>3. Government employee<br>4. Daily labor 5.Farmer 6.Other		
**Information of the index child**				
117.	Age of the young child (Months)	------------------		
118.	Sex of the young child	1. Female 2. Male		
119.	For how long did you breastfeed your child? (Months)	--------		
120.	What is his/her current breast feeding status?	1. Exclusive breast feeding<br>2. Partial breast feeding<br>3. Not breast feeding		
121.	At what age did the child start supplementary feeding/weaning food? (Months)	1. --------------------<br>2. I can't remember<br>3. still haven't started		
122.	Did the child receive measles vaccination (for those greater than nine months age)?	1. Yes<br>2. No		
123.	Did the child receive Rota virus vaccination? (Rvv1, Rvv2)	1. Yes<br>2. No		
124.	Does your child experience frequent and copious discharge of abnormally liquid faeces or diarrhea within the last two weeks?	1. Yes<br>2. No		
**Drinking water services**				
125.	What is the main source of drinking-water for members of your household?	1. Piped water<br>2. Protected well/spring<br>3. Unprotected sources(Unprotected well/spring/river)		

		4. Other (specify) _______________		
126.	Water always available from this source?	1. Yes 2. No		
127.	What is the main source of water used by members of your household for other purposes, such as cooking and hand washing?	1. Piped water<br>2. Protected well/spring<br>3. Unprotected sources(Unprotected well/spring/river)<br>4. Other (specify) ______________		
128.	Distance between drinking water sources and living home approximately? (Km)	------------------------		
129.	How long does it take to go there, get water, and come back?	---------------------		
130.	Daily requirements of waters in litters	--------------		
131.	Is the amount of water you get sufficient?	1. Yes 2. No		
132.	If Q131 is No, what was the (main) reason that you were unable to access sufficient quantities of water when needed?	1.water not available from source<br>2.water too expensive<br>3.source not accessible<br>4 Other (………………………..)		
133.	Storage of drinking water at home	1. Clay pots<br>2. Jerry can<br>3. Buckets<br>4. Others --------------		
134.	How often does your household clean the drinking water storage container?	1. Daily<br>2. Several times per week<br>3. Once a week<br>4. Once a month<br>5. Other (………………………..)		
135.	Where are the drinking water storage containers placed?	1. On the floor<br>2. Elevated above the floor<br>3. Others, specify		
136.	Do you cover the containers during storage?	1. Yes2. No		
137.	How is water taken from the drinking water containers?	1. Pouring<br>2. Dipping		
138.	Do you wash your hands before water collection?	1. Yes 2. No		
139.	Have you or any other household members done anything to this water to make it safer to drink?	1. Yes<br>2. No	If No, >>Q 142	
140.	What do you usually do to the water to make it safer to drink? Anything else?	1. Boil<br>2. Add bleach/chlorine<br>3. Use a water filter (ceramic, sand, composite, etc.)<br>4. Solar disinfection		
141.	When was the last time your household treated the water using this method?	1. Today<br>2. Yesterday<br>3. Less than one week ago<br>4. Don't know		
**Sanitation and hygiene facilities and practices**				
142.	Place of defecation	1. Ventilated improved [VIP] pit<br>2. Pit latrine with slab<br>3. Pit latrine without slab/open	If no facility , >>Q	

		pit<br>4. Bush or field (No facilities)	149	
143.	Do you share this facility with others who are not members of your household?	1. Yes<br>2. No		
144.	How many households use this latrine facility?	-----------		
145.	Do all household members use the facility?	1. yes always<br>2. yes sometime<br>3. no rarely<br>4. never		
146.	Where is this latrine facility located?	1. In own dwelling<br>2. In own yard / plot<br>3. Elsewhere		
147.	Is everyone in the household able to access and use the latrine at all times of the day and night (Adults)?	1. Yes<br>2. No	If Yes, >>Q 149	
148.	What was the (main) reason that household members were unable to access and use the toilet at all times of the day and night?	1. Unable to use the toilet<br>2. Unable to access the toilet<br>3. Toilet not safe to use<br>4. Other (specify)		
149.	Where do children under 5 from this household usually go to defecate?	1. household/public latrine<br>2. Open defecation<br>3. other (specify)-------------------		
150.	If there are children under 5 who don't use the latrine what is done with their feces?	1. Collected and disposed in latrine<br>2. Collected and disposed elsewhere<br>3. Buried it<br>5. Other---------------------------------<br>-		
151.	Would you kindly show me how your household disposes of used water?	1 Yes 2 No (refused)		
152.	What are the points of discharge of household's used water? (Observation)	1. Sanitation facility/pit<br>2. Open channel<br>3. Space outside premises<br>4. Premises' yard or garden<br>5. Other--------------------------		
153.	Where does your household dispose of domestic waste?	1. Household pit<br>2. Disposal within household yard/plot<br>3. Undesignated open area<br>4. Bury it<br>5. Burned		
154.	Does the latrine most often used have hand washing facilities with soap?	1. Yes, with soap and water<br>2. Sometimes<br>3. Take own soap and water<br>4. No<br>5. Other reason (specify) ______		
155.	What material do you use for anal cleansing after defecation?	1. Washes with water<br>2. Wipe with paper<br>3. Wipe with paper and washes with water<br>4. Leaf<br>5. others --------------------------		

156.	Do you wash your hands…?	✓ (Tick one)	Yes	No		
		before eating				
		before preparing food				
		before feeding a child or breastfeeding a baby				
		after visiting the latrine/after defecation				
		after cleaning a baby's bottom or disposing of a child's feces				

157.	Cleansing materials used to wash hands	1. Water and soap<br>2. Water and ash<br>3. only water

THANK YOU FOR YOUR HELP IN THIS RESEARCH
